# Granulomatosis (Wegener's granulomatosis) with polyangiitis presented as pulmonary manifestation: a case report

**DOI:** 10.1002/rcr2.674

**Published:** 2020-10-13

**Authors:** Qusay Jummaa Lazim, Sinan Shakir Gheni Atrah, Khalid Jawad Mutlag, Haider Saadoon Qasim Alhilfi, Ahmed Muhi Fahad, Ahmed Salih Alshewered

**Affiliations:** ^1^ Department of Cardiovascular Surgery Al‐Sadder Teaching Hospital, Misan Health Directorate, Ministry of Health/Environment Misan Iraq; ^2^ Department of Radiology Al‐Sadder Teaching Hospital, Misan Health Directorate, Ministry of Health/ Environment Misan Iraq; ^3^ Department of Rheumatology Al‐Sadder Teaching Hospital, Misan Health Directorate, Ministry of Health/ Environment Misan Iraq; ^4^ Faculty of Medicine, Department of Medicine Misan University Misan Iraq; ^5^ Misan Radiation Oncology Center Misan Health Directorate, Ministry of Health/Environment Misan Iraq

**Keywords:** Antineutrophil cytoplasmic antibody, granulomatosis, polyangiitis, pulmonary vasculitis, Wegener's granulomatosis

## Abstract

Pulmonary vasculitis can be the manifestation of several systemic illnesses such as primary systemic vasculitis, collagen vascular diseases, and systemic diseases associated with autoantibodies. It may be associated with granulomatous, eosinophilic, lymphoplasmacytic and neutrophilic inflammatory diseases. In this case report, we describe a 22‐year‐old female presented with intermittent fever, sweating and shivering, haemoptysis, sore throat, shortness of breath, fatigue, loss of appetite, nausea, non‐projectile vomiting, dizziness, and dark coloured urine. The diagnosis of granulomatosis with polyangiitis was made utilizing biochemical and radiological tests. Several pharmacological therapies were tried including rituximab. The patient made a good recovery and was discharged home after 12 days of hospitalization. The knowledge of the main radiographic and computed tomography (CT) scan findings, in association with clinical and laboratory data, often enables non‐invasive diagnosis of pulmonary vasculitis.

## Graphical abstract

The diagnosis of vasculitis is often delayed because several other disorders may mimic the same clinical manifestations; therefore, the knowledge of the main radiographic and high‐resolution computed tomography (HRCT) findings, in association with clinical, laboratory, and serum data, often enables non‐invasive diagnosis of pulmonary vasculitis.

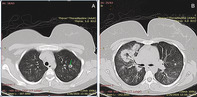

## Introduction

Pulmonary vasculitis is a non‐infectious inflammatory disorder that mainly affects the blood vessels of the lung, from the main pulmonary artery to alveolar capillaries, and encompass a clinically, radiologically, and histopathologically heterogeneous group associated with systemic vasculitis [1]. The most common radiographic abnormality is pulmonary nodules or consolidation with cavitation [2]. Less frequent radiological findings consist of nodules without cavitation, increased bronchovascular lines involving the lung parenchyma, mediastinal or hilar lymph node enlargement, and pleural effusion [3]. These radiological signs can be additional clues for correct and timely diagnosis [3].

## Case Report

A 22‐year‐old female presented with history of haemoptysis for eight days. The condition started as a productive cough (green sputum containing streaks of blood). Over the course of the following days, her haemoptysis worsened as she was coughing copious amount of blood. She also had constitutional symptoms (fatigue, anorexia, intermittent fever, sweating, and shivering at night), sore throat, shortness of breath, nausea, non‐projectile vomiting, dizziness, and dark coloured urine. The patient had no past medical and surgical history of note. She was not on any long‐term medications.

On examination, she was conscious, oriented, looked toxic, dyspnoeic, and pale. Her vital signs were as follows: temperature 38.5°C, heart rate 118 b/min, blood pressure 120/80 mmHg, respiratory rate 26 cycle per minute, and peripheral capillary oxygen saturation (SpO_2_) 89% on room air. There were petechiae in the roof of the oral cavity. Auscultation of the chest revealed bilateral bronchial breath sounds at midzone. The abdominal and pelvic examination was unremarkable. She had no pedal oedema. There was no cervical, axillary, or inguinal lymphadenopathy.

The following investigations were performed and their results as shown in Table 1.

**Table 1:** Investigations.

Investigations		After eight days of complaints	At sixth day of admission	At 11th day of admission	50 Days after completion of rituximab
CBC	HBG (g/dL)	10.2	8.1	11.1	13.2
	WBC (10^3^/μL)	11.4	14.4	13.4	10.8
	PLT (10^3^/μL)	378	256	312	214
APTT		32.0 sec	N/A	N/A	N/A
PT		21.8 sec	N/A	N/A	N/A
INR		2.1	N/A	N/A	N/A
ESR (mm/1 h)		98	57	27	17
Blood group		O positive			
Blood urea (mg/dL)		19.85	N/A	39	Normal
Serum creatinine (mg/dL)		0.39	N/A	0.4	Normal
Liver function test		Normal	Normal	Normal	Normal
Viral markers (HBV, HCV, HIV, VDRL)		Negative			
Sputum AFB for three consequential days		Negative			
Sputum for Gene‐Expert		Negative			
Sputum for culture and sensitivity		No pathogenic bacteria			
Sputum for cytological examination		Chronic granulomatous inflammation and no malignant cells could be seen			
Blood film morphology		Normochromic normocytic anaemia with mild leucocytosis			
CRP		Positive			
Latex test (RF)		Negative			
cANCA		124 (strong positive)			
pANCA		1.2			
Serum total protein (g/dL)		N/A	5.74	6.25	N/A
Serum albumin (g/dL)		N/A	3.04	3.5	N/A
LDH (U/L)		N/A	323	247	N/A
GUE		Unremarkable even there was no protein urea			

AFB, Acid fast Bacilli; ANCA, antineutrophil cytoplasmic antibody; APTT, activated partial thromboplastin time; CBC, complete blood count; CRP, C‐reactive protein; ESR, erythrocyte sedimentation rate; GUE, General Urine Examination; HBG, Hemoglobine; HBV, Hepatitis B Virus; HCV, Hepatitis C Virus; INR, international normalised ratio; LDH, lactate dehydrogenase; PLT, Platelets; PT, prothrombin time test; RF, rheumatoid arthritis factor; VDRL, Venereal Disease Research Laboratory test; WBC, white blood cell.

The patient was admitted to hospital. She was commenced on intravenous (i.v.) vancomycin as an empirical treatment for a possible chest infection given the clinical picture. During her stay, she developed right ankle joint pain and tenderness, and pitting oedema over both legs. On day 5, the temperature spiked to 40°C associated with rigors. The fever did not respond to parenteral paracetamol. As the patient was distressed due to high fever, we decided to administer dexamethasone. The following morning, a dramatic response was observed. Her fever subsided, haemoptysis improved, and the right ankle joint pain eased. The response to steroid and the failure of other treatments raised the possibility of a rheumatological disease causing this illness, like vasculitis. Therefore, we consulted one of the rheumatologist colleagues who shared the same suspicion around vasculitis. On the eighth day, she received two pints of whole blood, albumin infusion 100 mL (20 g)/day for three days, methylprednisolone vial 500 mg i.v. infusion/day for three days, omeprazole capsule 40 mg/day, and continued vancomycin injection.

On the 11th day, she continued to improve. At the point, she was fever free for six days and her dyspnoea as well as the joint pain subsided. She was coughing minute amount of blood.

**Figure 1:**
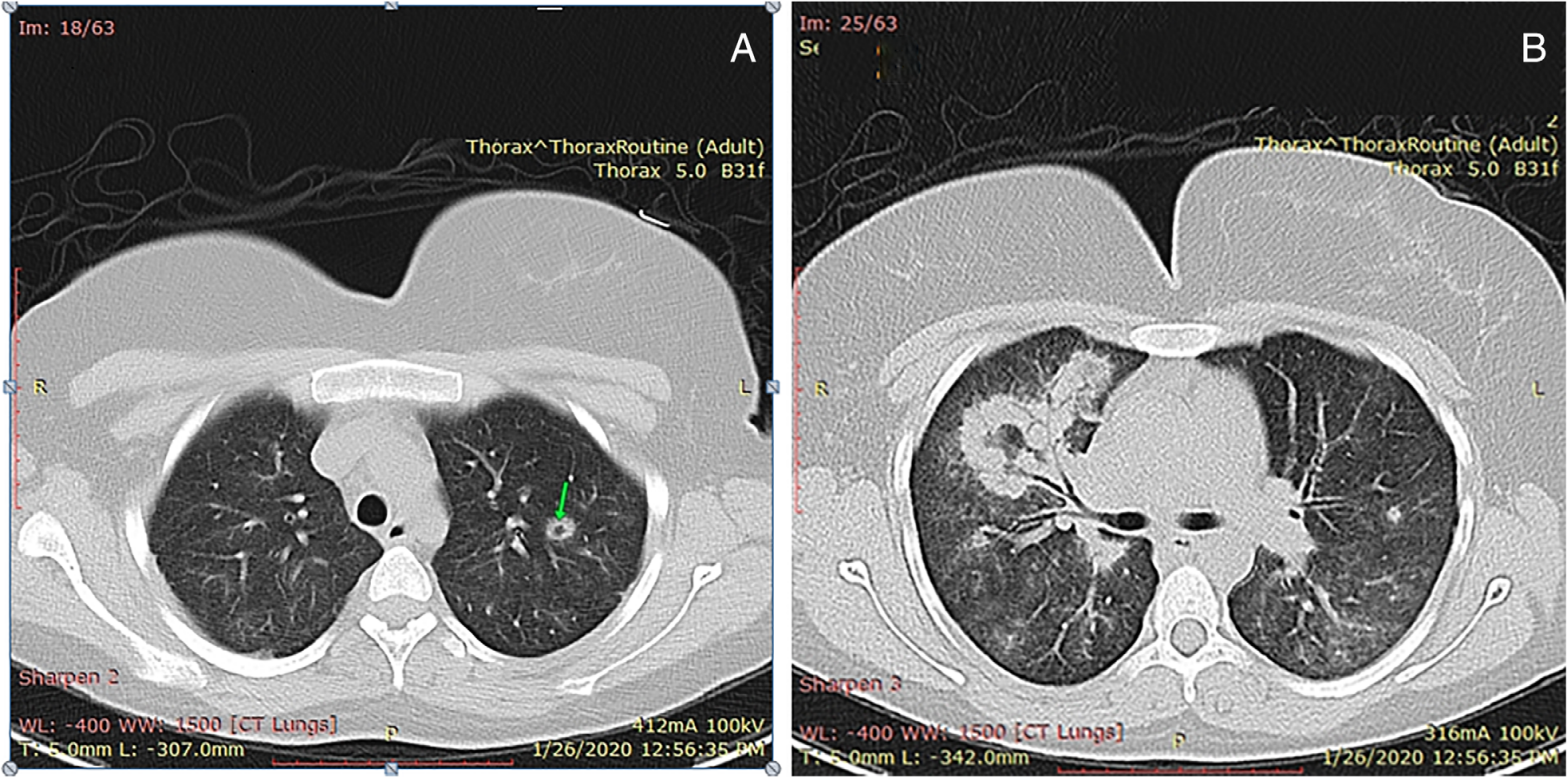
(A) Axial image of chest computed tomography (CT) scan showed a diffusely distributed pattern of centrilobular perivascular ground‐glass opacities and multiple nodular densities, in which one of them shows central cavitation (arrow). (B) CT scan revealed a segmental consolidation in the anterior segment of the right lung and irregular necrosis with multiple nodules and ground‐glass opacities in both lungs.

**Figure 2:**
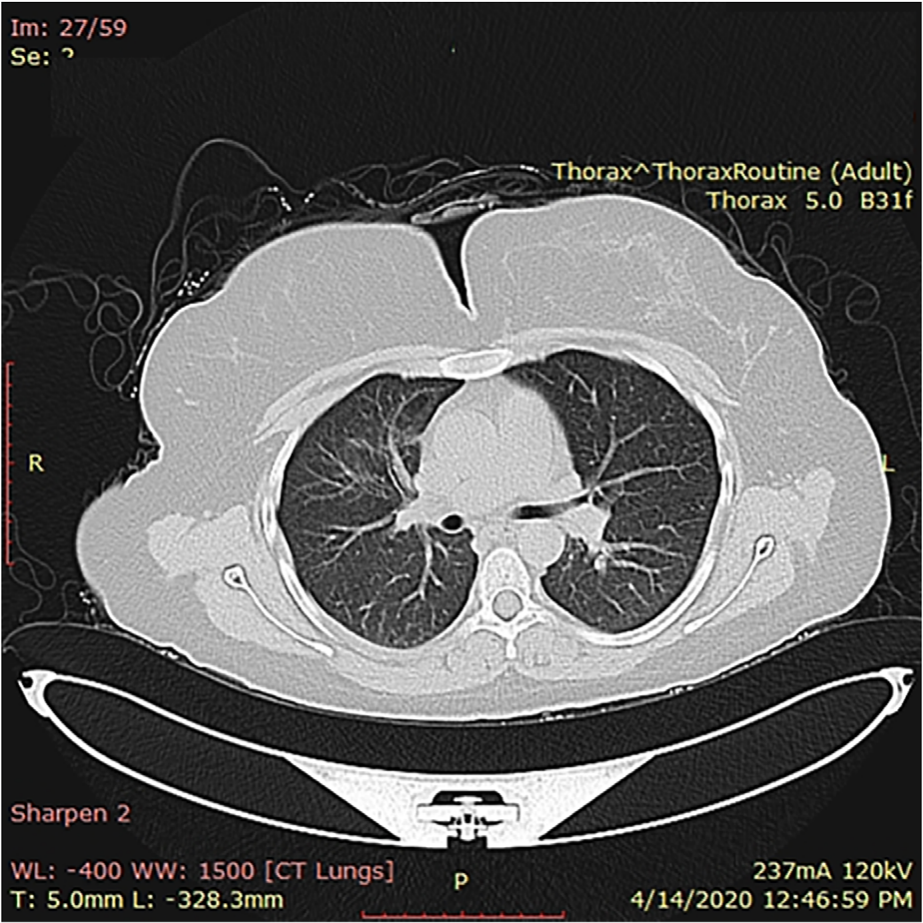
Chest computed tomography (CT) scan post treatment shows that the segmental consolidation, irregular necrosis, multiple nodules, and ground‐glass opacities in both lungs were disappeared.

On the 12th day, induction of remission regimen was planned through administrating high‐dose steroid at 50 mg prednisolone/day and rituximab injection 375 mg/m^2^ every week for four weeks. At the end of the course, the haemoptysis resolved and she was well enough to be. She developed moon face and acne vulgaris, but she was well apart from that. According to those investigation results (Table 1) and advice of the rheumatologist, the diagnosis of granulomatosis with polyangiitis was concluded. We advised her to have a follow‐up every month for next three months and then bimonthly for one year (Figs. 1, 2).

## Discussion

The clinical features presented in this case report were non‐specific and the provisional diagnoses were bronchopneumonia or pulmonary tuberculosis because they are common in our society. The pulmonary primary vasculitis is a rare condition, and it is often proved challenging to reach this diagnosis [4]. In this case, a multidisciplinary approach (internal medicine physician, radiologist, rheumatologist, and oncologist) was needed to reach the diagnosis. Laboratory test aided the diagnosis by antineutrophil cytoplasmic antibody (cANCA) positivity as well as the exclusion of malignancy and pulmonary tuberculosis through relevant tests. According to the study by Lee et al., the most common pattern on computed tomography (CT) images at the initial presentation is the presence of nodules or masses in 90% of patients [5]. The use of cytoplasmic ANCA is highly sensitive (90–95%) for the detection of active, systemic ANCA‐associated granulomatous vasculitis, and has a specificity of approximately 90% [6]. We attempted bronchoscopy to obtain a biopsy for confirmation of the diagnosis histopathologically; however, the procedure carried a risk due to bleeding from nasal mucosa, so it was abandoned. We also felt that needle lung biopsy would be hazardous. Induction of remission in our patient was achieved by rituximab with high‐dose glucocorticoids as shown in the results of the randomized RAVE4 and RITUXVAS5 trials, which demonstrated that rituximab‐based regimen was as effective and safe as conventional cyclophosphamide to induce remission, but there was no maintenance therapy prescribed [7].

The diagnosis of vasculitis is often delayed because several other disorders may mimic the same clinical manifestations; therefore, the knowledge of the main CT findings, in association with clinical, laboratory, and serum data, often enables non‐invasive diagnosis of pulmonary vasculitis.

### Disclosure Statement

Appropriate written informed consent was obtained for publication of this case report and accompanying images.
